# Lemierre's Syndrome: A Rare Case of Pulmonic Valve Vegetation

**DOI:** 10.1155/2013/519720

**Published:** 2013-03-21

**Authors:** Clara Kwan, Lou Mastrine, Manfred Moskovits

**Affiliations:** ^1^Department of Medicine, Maimonides Medical Center, 4802 Tenth Avenue, Brooklyn, NY 11219, USA; ^2^Cardiology, Department of Medicine, Maimonides Medical Center, 4802 Tenth Avenue, Brooklyn, NY 11219, USA

## Abstract

Lemierre's syndrome is an uncommon complication of pharyngitis commonly associated with an anaerobic gram negative bacterium, *Fusobacterium necrophorum*. The syndrome usually affects young healthy adults with the mean age of 20 and is characterized by recent pharyngitis followed by ipsilateral internal jugular vein thrombosis and septic thromboembolism. The treatment is at least 6 weeks of antibiotics; the role of anticoagulation is unclear. The following presentation is a case of Lemierre's syndrome in a 23-year-old healthy individual who is infected by a rare species: *Fusobacterium nucleatum*. The case is complicated by septic emboli to the lungs and impressive seeding vegetation to the right ventricular outflow tract (RVOT) at the pulmonic valve of the heart.

## 1. Introduction

A 23-year-old male presented to the emergency room (ER) with a chief complaint of chest pain. The pain was described as midsternal, sharp, graded 8/10, worse with inspiration, associated with shortness of breath, palpitations, and decreased exercise tolerance. Further, he reported viral symptoms including headache, vomiting, fever of 103 Fahrenheit, and night sweats for 7 days prior to ER arrival. Prior to his symptoms, he reports celebrating with friends for several days, including frequent alcohol intake and cigarette smoking.

The patient reports a penicillin allergy, with a reaction of “swelling.” His family history is noncontributory. He is a college student. He is a former cigarette smoker; smoking half a pack a day for 5 years and smokes marijuana weekly. He also admits to heavy alcohol use.

On physical exam, the patient was normotensive but tachycardic with a heart rate of 120–140 beats per minute. He appeared anxious. There was no jugular venous distention on the neck exam, no bruits, and no lymphadenopathy. His chest was symmetric with poor inspiratory effort and basilar crackles bilaterally. The cardiovascular exam revealed no murmurs, rubs, or gallops. All other system examinations were normal.

On admission, laboratory investigations showed a white blood count of 6.8 × 10^9^/L, hemoglobin of 11.4 g/dL, hematocrit of 33%, and platelets of 33 × 10^9^/L. Chest X-ray showed a possible left lower lobe infiltrate. Cardiac enzymes (CEs) and two sets of blood cultures were obtained. Bedside transthoracic echocardiogram showed a severely decreased ejection fraction of 30% with global left ventricular hypokinesis and no right ventricular involvement.

Myocarditis was originally suspected. The patient was admitted to the coronary care unit (CCU) and was placed on carvedilol 6.25 mg by mouth (po) every (q) 12 hours (h) and enalapril 2.5 mg po daily as cardiac protectants as well as Motrin and Toradol for chest pain. Serial CEs were within normal limits, and myocarditis was ruled out. Blood cultures revealed gram negative bacteremia, infectious disease services were consulted, and broad-spectrum antibiotics were administered including aztreonam 2000 mg IV q8h and gentamycin 80 mg IV q12h. As previously mentioned, the patient had thrombocytopenia with platelets of 33 × 10^9^/L and giant platelets seen on a peripheral blood smear. Hematology/Oncology suggested that it was secondary to gram negative sepsis.

**Figure 1:**
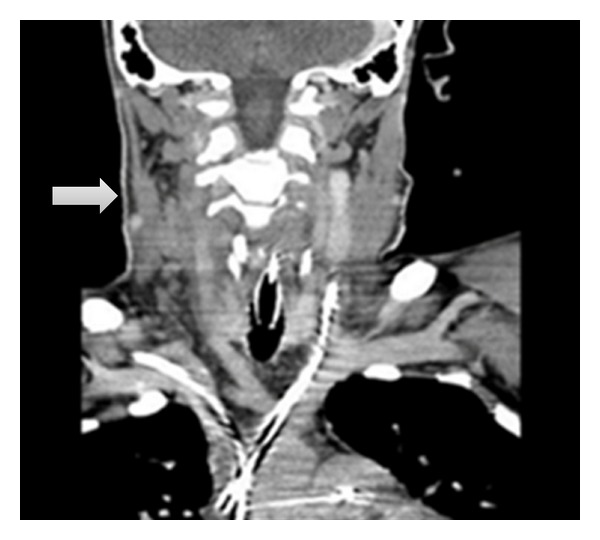
CT head shows a nonenhancing structure consistent wtih DVT at the right IJ vein (arrow).

The patient became hypotensive on day 3, and a right heart catheterization was performed which confirmed suspicion of septic shock. Central venous access for intravenous (IV) pressor therapy was also desired; upon attempted internal jugular (IJ) vein cannulation, pus-like material was aspirated. A bedside ultrasound showed bilateral cystic structures resembling abscesses. Computed tomography (CT) of the neck/chest/abdomen/pelvis was performed revealing a right parapharyngeal soft tissue structure compatible with abscess in the oropharynx. Moreover, lung fields showed septic pulmonary emboli bilaterally. Otolaryngology was consulted for the abscess and recommended continuing antibiotics. A venous duplex scan was positive for an acute deep venous thrombosis (DVT) in the right IJ vein also seen in the CT scan (Figure 1). Enoxaparin was started for DVT. The patient deteriorated on day 4 with persistent fever, hypotension, and hypoxemic respiratory failure requiring intubation and IV pressor therapy. Three blood cultures were growing gram negative rods of *Fusobacterium nucleatum* and gram positive group C *Streptococcus*. Polymixin B 1000000 units IV q12h, vancomycin 1000 mg IV q12h, and rifampin 300 mg IV q12h were added to his current antibiotic regimen.

**Figure 2:**
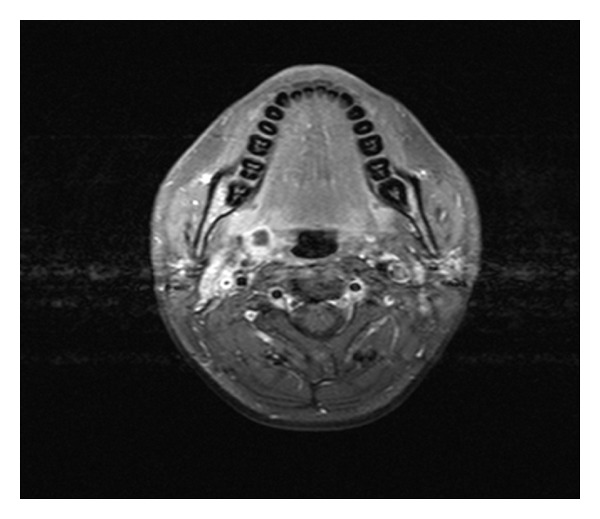
MRI head shows an enhancing structure, an abscess on the right side measured 1.3 × 1.5 × 1.3 cm, and also a DVT in the IJ vein more posterior and lateral to the abscess.

**Figure 3:**
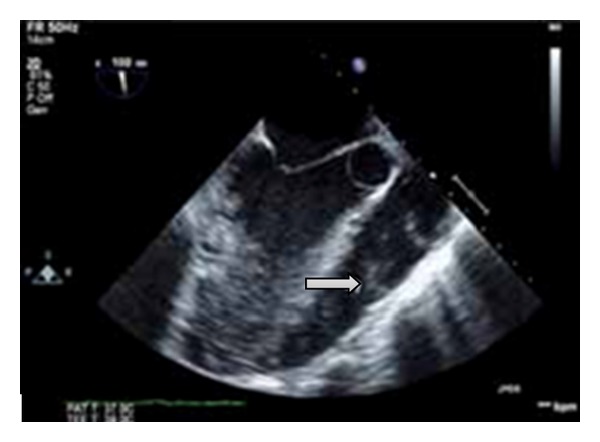
Echocardiogram shows a mass on the RVOT with association to the pulmonic valve (arrow).

**Figure 4:**
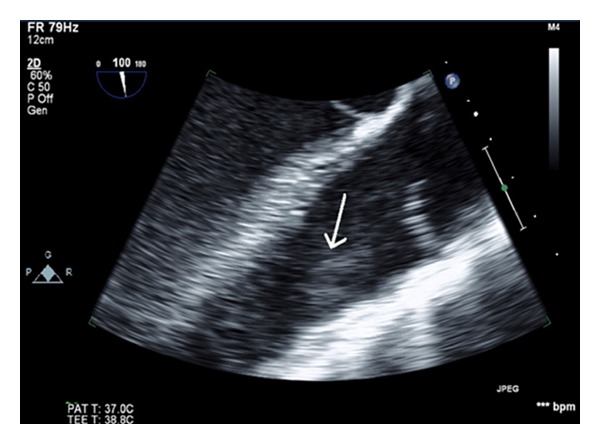
Transesophageal echocardiogram showing a pulsatile mass at the pulmonic valve.

On day 6, the patient's symptoms improved and he was extubated successfully. MRI of the neck showed a parapharyngeal abscess measured 1.3 × 1.5 × 1.3 cm (Figure 2) and a thrombus in the right IJ vein. Transesophageal echocardiogram (TEE) was performed and showed an impressive pulsatile mass on the right ventricular outflow tract (RVOT) with association to the pulmonic valve (Figures 3 and 4). With the presentation of pharyngeal abscess, right IJ DVT, blood cultures growing *Fusobacterium*, septic pulmonary embolism, and infective endocarditis at the pulmonic valve, a diagnosis of Lemierre's disease was made on day 9. A repeat TEE on day 12 showed that the RVOT vegetation had significantly regressed, and a repeat venous doppler study showed a small residual thrombus at the right IJ vein. A peripherally inserted central venous catheter was placed for long-term antibiotic for 4–6 weeks, and long-term anticoagulation with coumadin was started. Repeated MRI also showed interval resolution of the parapharyngeal abscess and he was discharged home on day 21 in stable condition.

## 2. Discussion

Lemierre's syndrome is characterized by pharyngitis, fevers, IJ vein thrombosis, and septic embolic disease [1]. It is often found in healthy adolescents or young adults [1, 2]. The most common pathogen in this syndrome is *Fusobacterium*, particularly *F. necrophorum*, a gram negative anaerobic bacillus. *F. necrophorum* is a commonly identified pathogen that is part of the normal flora of the head and oropharynx. It has been postulated that a precipitating condition, such as pharyngitis or sinusitis can lead to invasive disease [2]. About 57% of case reports were caused by *F. necrophorum,* another 30% were caused by other *Fusobacterium *species, and 3% were caused by *F. nucleatum *[2]. The bacteria identified in this case, *F. nucleatum*, is a bacterium commonly found in the oropharynx and has the ability to aggregate to other anaerobes and secretes enzymes to cause plaque in teeth. Lemierre's syndrome can also be caused by other anaerobic or nonanaerobic bacteria, such as *Bacteroides* and *Staphylococcus* [2, 3]. Lemierre's syndrome can be precipitated by dental procedures, intravenous drug abuse, infectious mononucleosis, and possibly smoking [3]. The actual pathogenesis of Lemierre's disease is still unclear. Based on our patient's history of present illness, we believe poor oral hygiene with alcohol use and cigarette smoking promoted his infection.

## 3. Treatment Option

The predominant treatment is often beta-lactamase inhibitor such as ampicillin/sulbactam or a beta-lactam plus metronidazole [1, 2, 4]. In some cases, clindamycin is also used as it has adequate coverage for methicillin-resistant *staphylococcus aureus*. In this case, the blood cultures were found to have both gram negative *F. nucleatum* and gram positive group C *Streptococcus*. Additionally, the patient is allergic to penicillin; therefore, moxifloxacin and metronidazole were used to cover polymicrobial and anaerobic infections.

## 4. Rare Vegetation

One of the most unique findings here is the vegetation at the RVOT and pulmonic valve. Right-sided involvement in endocarditis is generally uncommon, making up approximately 5% of all cases found yearly. Many of the examples seen in patients with right-sided endocarditis are intravenous drug users, patients with congenital heart disease, pace makers, and central venous lines [5–7].

## 5. Anticoagulation

Anticoagulation in Lemierre's disease is controversial. Their role has only been observed in case series; no controlled studies assessing their effectiveness in thrombophlebitis of the jugular system have been done. Routine systemic anticoagulation for jugular vein thrombosis may promote early resolution of thrombophlebitis and bacteremia, preventing spread of disease [8, 9]. Some physicians recommend anticoagulation in cases complicated by cerebral infarcts, complete thrombosis of the jugular vein, or thrombosis extending into the cavernous sinus [1, 2, 10]. The optimal duration of treatment is unknown and should be evaluated from case to case depending on the extent of the thrombus and the location [10]. Our patient was treated with enoxaparin and coumadin immediately following the diagnosis of DVT, and reduction of size was seen on repeat venous duplex study one week later. He was treated with oral anticoagulation until discharge and scheduled for office visits at several of our outpatient clinics.
